# Supplementary material for: Multi‐dataset identification of innovative feature genes and molecular mechanisms in keratoconus
Source: J Cell Mol Med. 2024 Sep 19;28(18):e70079. doi: 10.1111/jcmm.70079 (PMC11412914; doi:10.1111/jcmm.70079)
Supplement: Supplementary file 2 — Material S2. [file JCMM-28-e70079-s002.zip › 7.GSEA analysis/6.SNORA74B/KEGG_PHOSPHATIDYLINOSITOL_SIGNALING_SYSTEM.html]

Details for gene set KEGG\_PHOSPHATIDYLINOSITOL\_SIGNALING\_SYSTEM[GSEA]

|  || Dataset | SNORA74B.group.cls#h\_versus\_l |
| Phenotype | group.cls#h\_versus\_l |
| Upregulated in class | l |
| GeneSet | KEGG\_PHOSPHATIDYLINOSITOL\_SIGNALING\_SYSTEM |
| Enrichment Score (ES) | -0.31510895 |
| Normalized Enrichment Score (NES) | -1.3454309 |
| Nominal p-value | 0.12922466 |
| FDR q-value | 0.31517255 |
| FWER p-Value | 0.931 |
Table: GSEA Results Summary

  

Fig 1: Enrichment plot: KEGG\_PHOSPHATIDYLINOSITOL\_SIGNALING\_SYSTEM      
 Profile of the Running ES Score & Positions of GeneSet Members on the Rank Ordered List

  

| SYMBOL | TITLE | RANK IN GENE LIST | RANK METRIC SCORE | RUNNING ES | CORE ENRICHMENT || 1 | PIK3R5 | na | 170 | 0.800 | 0.0631 | No |
| 2 | PRKCG | na | 2539 | 0.330 | 0.0228 | No |
| 3 | INPP4B | na | 2598 | 0.326 | 0.0488 | No |
| 4 | DGKH | na | 3893 | 0.263 | 0.0338 | No |
| 5 | DGKB | na | 4410 | 0.246 | 0.0398 | No |
| 6 | DGKZ | na | 5768 | 0.200 | 0.0176 | No |
| 7 | INPP5D | na | 6064 | 0.190 | 0.0253 | No |
| 8 | CALML6 | na | 7151 | 0.158 | 0.0073 | No |
| 9 | SYNJ1 | na | 7655 | 0.145 | 0.0051 | No |
| 10 | PIK3R1 | na | 8116 | 0.133 | 0.0031 | No |
| 11 | DGKD | na | 8196 | 0.131 | 0.0120 | No |
| 12 | PLCG2 | na | 8239 | 0.131 | 0.0219 | No |
| 13 | CALML5 | na | 8469 | 0.125 | 0.0259 | No |
| 14 | ITPR2 | na | 8911 | 0.116 | 0.0230 | No |
| 15 | PLCB3 | na | 9334 | 0.106 | 0.0199 | No |
| 16 | PLCB2 | na | 9573 | 0.101 | 0.0216 | No |
| 17 | PLCG1 | na | 9672 | 0.099 | 0.0272 | No |
| 18 | PIKFYVE | na | 10605 | 0.081 | 0.0072 | No |
| 19 | INPP1 | na | 10748 | 0.079 | 0.0098 | No |
| 20 | IPPK | na | 11091 | 0.073 | 0.0062 | No |
| 21 | DGKQ | na | 11280 | 0.070 | 0.0067 | No |
| 22 | PIP4K2B | na | 11358 | 0.069 | 0.0104 | No |
| 23 | PLCD3 | na | 12496 | 0.053 | -0.0179 | No |
| 24 | CALM2 | na | 12514 | 0.053 | -0.0139 | No |
| 25 | PTEN | na | 12756 | 0.050 | -0.0167 | No |
| 26 | PIK3R2 | na | 12946 | 0.047 | -0.0181 | No |
| 27 | DGKE | na | 12999 | 0.047 | -0.0156 | No |
| 28 | PIK3CA | na | 13079 | 0.046 | -0.0140 | No |
| 29 | PLCD1 | na | 13448 | 0.042 | -0.0210 | No |
| 30 | INPP5J | na | 14023 | 0.035 | -0.0345 | No |
| 31 | CALML3 | na | 15332 | 0.021 | -0.0705 | No |
| 32 | PIK3CB | na | 15769 | 0.017 | -0.0816 | No |
| 33 | INPP5K | na | 15823 | 0.016 | -0.0818 | No |
| 34 | ITPR3 | na | 16729 | 0.007 | -0.1073 | No |
| 35 | PLCB4 | na | 16838 | 0.006 | -0.1099 | No |
| 36 | PIK3C2B | na | 16971 | 0.005 | -0.1133 | No |
| 37 | INPP5E | na | 18387 | -0.011 | -0.1532 | No |
| 38 | PIP5K1C | na | 18480 | -0.012 | -0.1549 | No |
| 39 | PIK3CG | na | 18884 | -0.016 | -0.1651 | No |
| 40 | IMPA1 | na | 19024 | -0.017 | -0.1677 | No |
| 41 | PLCZ1 | na | 19726 | -0.026 | -0.1857 | No |
| 42 | PIK3C2A | na | 20182 | -0.030 | -0.1963 | No |
| 43 | INPPL1 | na | 20210 | -0.031 | -0.1945 | No |
| 44 | PIK3CD | na | 20354 | -0.032 | -0.1959 | No |
| 45 | INPP5A | na | 21505 | -0.046 | -0.2252 | No |
| 46 | CDS2 | na | 21680 | -0.048 | -0.2261 | No |
| 47 | PRKCA | na | 22239 | -0.055 | -0.2376 | No |
| 48 | CALM1 | na | 22340 | -0.056 | -0.2357 | No |
| 49 | PIK3C3 | na | 22677 | -0.061 | -0.2403 | No |
| 50 | CDS1 | na | 22852 | -0.063 | -0.2399 | No |
| 51 | DGKG | na | 23168 | -0.068 | -0.2433 | No |
| 52 | CALM3 | na | 23658 | -0.075 | -0.2510 | No |
| 53 | ITPR1 | na | 23722 | -0.077 | -0.2463 | No |
| 54 | DGKA | na | 24289 | -0.085 | -0.2554 | No |
| 55 | CDIPT | na | 24408 | -0.088 | -0.2513 | No |
| 56 | SYNJ2 | na | 24669 | -0.092 | -0.2510 | No |
| 57 | PI4KB | na | 25039 | -0.099 | -0.2532 | No |
| 58 | OCRL | na | 25177 | -0.102 | -0.2485 | No |
| 59 | PIP4K2C | na | 25967 | -0.116 | -0.2614 | No |
| 60 | INPP4A | na | 26031 | -0.117 | -0.2533 | No |
| 61 | PLCD4 | na | 26111 | -0.119 | -0.2454 | No |
| 62 | PI4KA | na | 26267 | -0.122 | -0.2395 | No |
| 63 | ITPKA | na | 26318 | -0.123 | -0.2305 | No |
| 64 | ITPK1 | na | 26769 | -0.134 | -0.2321 | No |
| 65 | PIP4K2A | na | 26936 | -0.138 | -0.2251 | No |
| 66 | IMPA2 | na | 27158 | -0.143 | -0.2194 | No |
| 67 | INPP5B | na | 27989 | -0.164 | -0.2294 | No |
| 68 | PIK3C2G | na | 28884 | -0.191 | -0.2389 | No |
| 69 | PIK3R3 | na | 29453 | -0.208 | -0.2376 | No |
| 70 | ITPKB | na | 30431 | -0.243 | -0.2452 | No |
| 71 | PLCB1 | na | 32854 | -0.381 | -0.2828 | Yes |
| 72 | PIP5K1A | na | 33089 | -0.403 | -0.2553 | Yes |
| 73 | PRKCB | na | 33882 | -0.508 | -0.2350 | Yes |
| 74 | DGKI | na | 34514 | -0.707 | -0.1932 | Yes |
| 75 | PLCE1 | na | 34694 | -0.990 | -0.1143 | Yes |
| 76 | PIP5K1B | na | 34724 | -1.356 | 0.0001 | Yes |
Table: GSEA details [plain text format]

  

Fig 2: KEGG\_PHOSPHATIDYLINOSITOL\_SIGNALING\_SYSTEM      
 Blue-Pink O' Gram in the Space of the Analyzed GeneSet

  

Fig 3: KEGG\_PHOSPHATIDYLINOSITOL\_SIGNALING\_SYSTEM: Random ES distribution      
 Gene set null distribution of ES for **KEGG\_PHOSPHATIDYLINOSITOL\_SIGNALING\_SYSTEM**
